# Maintaining overall cognitive and physical functioning in at‐risk older adults: study design of the MOVE project

**DOI:** 10.1002/alz70858_100762

**Published:** 2025-12-25

**Authors:** Costanza Papagno, Jorge Jovicich, Alessandra Dodich

**Affiliations:** ^1^ Center for Mind/Brain Sciences, University of Trento, Rovereto, TN, Italy; ^2^ Center for Mind Brain Sciences, University of Trento, Rovereto, TN, Italy; ^3^ University of Trento, Rovereto, Trento, Italy

## Abstract

**Background:**

Despite robust evidence supporting the link between falls and cognitive decline, the precise mechanisms underpinning this association remain elusive, as does the potential efficacy of targeted interventions to mitigate these risk factors. To address these knowledge gaps, the MOVE project aims to evaluate the differential effects of two interventions in modifying cognitive (i.e., prospective memory) and physical (i.e., risk of falls) decline trajectories, as well as aspects of intervention‐induced neural plasticity through advanced structural, functional and metabolic MRI indices.

**Method:**

The population of interest is represented by individuals at risk of cognitive decline enrolled from the general population (i.e. individuals with pre‐mild cognitive impairment or subjective cognitive decline). Ninety participants will be randomly assigned to a physical (MOT, *n* = 30) or cognitive intervention (COG, *n* = 30), or to a control group (WAIT, *n* = 30). Clinical and neuropsychological data, as well as lifestyle and motor measures, will be collected at baseline and at the end of the intervention by staff blinded to the subject assigned treatment. Primary outcomes are represented by post‐pre changes in a measure of prospective memory and in a fall risk prevention test, as well as changes in brain morphometry. Exploratory outcomes will include post‐intervention changes in a measure of subjective cognitive decline, loneliness and in metrics of cerebral tissue microstructure

**Result:**

We expect that the group receiving the MOT compared to the WAIT and COG groups will show a significantly lower risk of falls assessed through the fall risk prevention test at post‐intervention evaluation. On the other hand, we expect in the COG group a better performance in an ad hoc test developed to evaluate prospective memory compared to the other groups. However, considering the association between cognitive decline and risk of falls, crossed secondary effects of training could be observed

**Conclusion:**

The project will provide novel data on possible modulation of cognitive and physical status under the effect of different interventions, also unraveling the neural correlates, thus opening new avenues in defining preventive interventions in aging.

“Finanziato dall'Unione europea ‐ NextGenerationEU ‐ PNRR Missione 4 Componente 2 Investimento 1.3 ‐ Bando a Cascata AGE‐IT MOVE ‐ PE00000015 CUP B83C22004880006”

